# Thermoluminescence Characteristics of Alpha/Gamma Irradiated‐Aluminum Nitride

**DOI:** 10.1002/bio.70170

**Published:** 2025-04-11

**Authors:** Rodrigo Martinez‐Baltezar, E. F. Huerta, U. Caldiño, Emma Cortés‐Ortiz, Juan Azorín‐Nieto

**Affiliations:** ^1^ Departamento de Física Universidad Autónoma Metropolitana‐Iztapalapa Ciudad de México México; ^2^ Universidad Tecnológica de México‐UNITEC MÉXICO‐Campus Los Reyes México City México

**Keywords:** complex defects, deconvolution, fading, half‐lives, second derivative

## Abstract

Aluminum nitride doped with unintentional impurities was synthesized using the NH₄Cl(s)‐assisted vapor‐phase reaction method. X‐ray diffraction (XRD) confirmed the formation of the hexagonal wurtzite phase with lattice parameters **a** = 3.111 Å and **c** = 3.978 Å. Energy‐dispersive spectroscopy (EDS) detected the presence of Al, N, C, O, Si, and Fe in the aluminum nitride. Fourier‐transform infrared spectroscopy (FTIR) and Raman spectroscopy confirmed characteristic vibrational modes, further supporting the crystalline structure. Photoluminescence (PL) analysis revealed two excitation peaks at 280 and 335 nm, associated with C_N_Si_Al_, V_Al_3O_N_, and C_N_V_N_ complex defects. The emission spectrum exhibited a predominant peak at 405 nm, in agreement with previous reports, suggesting a correlation with V_Al_2O_N_ defects. Thermoluminescence (TL) measurements showed glow curves with peaks at 460, 525, and 600 K, confirmed through second derivative and deconvolution analysis. Activation energy values (0.69 and 0.45 eV) align with those reported for Si_Al_ defects. The TL response displayed saturation at approximately 140 Gy, with the third peak exhibiting a linear response in the range of 12.6–136 Gy. These results highlight the potential of AlN for TL applications, emphasizing the role of unintentional impurities in defect formation and luminescence properties.

## Graphical abstract

AlN doped with unintentional impurities was synthesized via NH₄Cl‐assisted vapor‐phase reaction. Structural and optical results show defect‐related luminescence, with thermoluminescence peaks at 460, 525, and 600 K. The findings highlight the role of point defects in the luminescence properties of AlN, demonstrating its potential for dosimetric applications.

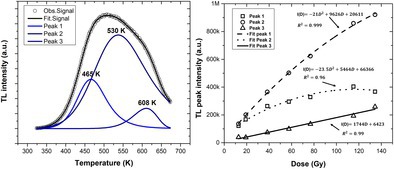

## 1 Introduction

Aluminum nitride (AlN) is a wide band gap material (6.2 eV) with a wurtzite crystal structure and covalent bonding [1]. Its unique properties, including high thermal conductivity, low thermal expansion coefficient, high electrical resistivity, excellent mechanical strength, and good chemical stability, make it highly attractive for applications based on luminescence phenomena [2, 3]. Due to its exceptional thermal conductivity (320WK/m at 300K) and high electrical resistivity ($\rho =10^{11}\;W.m$), AlN is widely used as a heat sink in electronic and optoelectronic devices [4, 5]. Furthermore, AlN exhibits PL emissions in the UV‐blue spectral region under UV irradiation, a characteristic that has become a growing interest in its potential applications in radiation dosimetry [1, 6, 7]. These properties make AlN a promising material for applications in radiation dosimetry [8, 9]. However, a significant challenge for its practical use in dosimetric applications is its high fading at room temperature, as reported by several authors [7, 8, 9, 10, 11].

Trinkler et al. investigated TL properties of AlN:Y₂O₃ under diverse radiation sources, reporting that this material exhibits a broad glow curve ranging up to 500°C, with a peak varying from 200 to 300°C (depending on the heating rate), which is associated with a PL emission at 400 nm [9]. Yanagida et al. reported that the glow curve of AlN exhibited two TL peaks centered at 80 and 320°C, which are related to PL emissions centered at 340 nm and another at a longer wavelength, respectively [10].

In another study, Trinkler et al. investigated the TL properties of AlN with oxygen ion implantation, reporting that this material exhibits two TL peaks centered at 80 and 220°C [11]. Onoda et al. investigated the TL properties of Eu‐doped AlN, finding that this material exhibits seven TL peaks [7]. The sensitivity and TL response as a function of dose for each peak depends on Eu concentration [7]. Kojima et al. suggested that SrF incorporation into the AlN lattice suppresses electron trapping for shallow centers in AlN; as a result, charge carriers are only captured by deeper trapping centers [1]. These researchers suggest that the TL and PL AlN properties depend on specific impurities and impurity concentrations in the AlN lattice. This observation encourages further research into the TL properties of AlN with other impurities.

Carbon (C), oxygen (O), and silicon (Si) are the main unintentional impurities reported by several authors when studying the PL properties of AlN [4, 6, 12]. However, only a few studies have investigated the thermoluminescence properties of this material [8, 9].

This paper reports the results of studying the TL characteristics and dosimetric properties of alpha/gamma irradiated‐AlN doped with carbon, oxygen, silicon, and iron (AlN: C, O, Si, Fe), synthesized using the NH₄Cl‐assisted vapor phase reaction method. The TL glow curve shows three peaks at 465, 530, and 608 K. The TL kinetic parameters and the half‐life of each peak were analyzed using the deconvolution method based on the general order kinetics model (GOK). The results show that 465 and 530 K peaks have short half‐lives (< 3 days), while the third peak has a half‐life of 522.4 years. The deconvolution method was also used to study TL intensity as a function of dose for each glow peak. The results show that for both the first and second peaks, TL intensity versus dose exhibits nonlinear behavior, while the third peak fits a linear behavior. These results suggest that the third peak has promising properties for applications in TL dosimetry.

## 2 Materials and Methods

### 2.1 Synthesis

Al (s) (Aldrich 99.9%) and NH_4_Cl (s) (Aldrich 99.5%) were mixed at a weight ratio of 3:2 in a corundum mortar and placed in the center of a quartz tube. This mixture was dried at 130°C for 2 h in the center of a tubular furnace (Thermolyne F21135) under 10 L/min constant flow of N_2_ (g). The temperature was raised to 1000°C, which induced a series of chemical reactions as described by Zheng et al. [3]. During this stage, the N_2_ (g) flux was kept constant. After 3 h, the system was allowed to cool to room temperature, and the resulting powder was scraped off the crucible.

### 2.2 Structural and Elemental Characterization

The crystalline structure of the synthesized product was determined by Bragg–Brentano X‐ray diffraction (XRD) using a Bruker D‐8 Advance diffractometer equipped with a graphite monochromator on the diffracted beam and a Cu Kα radiation source (*λ* = 0.15418 nm), operating at 40 kV and 35 mA. A nickel filter was employed to remove the Cu Kα₂ component. The morphology was studied using a JEOL JSM‐6390LV scanning electron microscope (SEM) at an acceleration voltage of 20 kV. The elemental composition was determined by energy‐dispersive X‐ray spectroscopy (EDS), employing a Pentafet Oxford Si‐Li detector integrated into the SEM. The characteristic vibrational modes were investigated by Fourier transform infrared (FTIR) spectroscopy using a Perkin‐Elmer GX‐2000 system equipped with an attenuated total reflectance (ATR) unit (Smith diamond Duran Sample II). The FTIR spectrum was recorded over 64 scans, ranging from 2000 to 400 cm^−1^, with a resolution of 4 cm^−1^. The characteristic vibrational modes were also investigated by Raman spectroscopy using a Micro Raman System RM3000 spectrometer equipped with an excitation laser of 532nm wavelength. The laser light was focused on the samples by a 100× objective‐equipped microscope. The Raman spectrum was recorded from 1500 to 0 cm^−1^.

### 2.3 Photoluminescence

PL spectra were measured using a Horiba Jobin‐Yvon Fluorolog 3‐22 spectrofluorometer equipped with a 150W ozone‐free Xe lamp. The excitation spectrum was recorded with an emission wavelength at 409 nm, in the spectral range of 250–380 nm, and the emission spectrum was measured with an excitation wavelength at 330 nm, in the spectral range of 360–600 nm.

### 2.4 Thermoluminescence

The TL response as a function of dose was obtained by irradiating the samples at different doses in the range of 2.4–134.4 Gy, using an ^241^Am source. TL glow curve was registered using a Harshaw 3500TL analyzer (Thermo Scientific, USA) at a heating rate of 2°C/s from 50°C to 400°C under a N_2_ atmosphere to reduce thermal noise from the heating planchet of the TL reader. To ensure repeatability, this process was repeated five times.

Kinetic parameters were determined by the deconvolution method using the “tgcd” open‐source package in R. The half‐life for each TL component in the glow curve was calculated based on Eq. (15) and the deconvolution results. The TL response for each peak as a function of dose was analyzed using the software OriginPro version 9.0.

### 2.5 Deconvolution Details

The “tgcd” is an open‐source R package developed by Peng et al. [13] and improved by Peng et al. [14]. This package utilizes some expressions from the GOK model, semi‐analytical expressions from the one‐trap, one‐recombination‐center model (OTOR), and the modified Levenberg–Marquardt Levenberg–Marquardt algorithm to deconvolute TL curves.

To determine TL kinetics parameters, the GOK model (referred to as g1 in the “tgcd” package) was employed, given that it is the most used model by researchers in this field. Kitis et al. developed the analytical approximations for the GOK model [15]. This section outlines the key points of this derivation. The GOK equation for TL intensity is [15]

(1) $$\begin{array}{c} I\left(T\right)=s^{\prime \prime }n_{0}^{b}\exp\left(-\frac{E}{\mathrm{kT}}\right){\left(1+\frac{\left(b-1\right)s^{\prime \prime }n_{0}^{b-1}}{q}\int_{T_{i}}^{T}\exp\left(-\frac{E}{kT^{\prime }}\right)dT^{\prime }\right)}^{\frac{b}{1-b}} \end{array}$$

where *b* is the kinetic order, *E* is the activation energy, *n*_0_ is the initial number of trapped electrons, *q* is the heating rate, and $s^{”}$ is defined as

(2) $$s^{”}=\frac{s}{N^{b-1}},$$

where *s* is the frequency factor and *N* is the total number of electron traps in the crystal. The expression reaches its maximum at *T*_*M*_, as given by the following equation:

(3) $$\begin{array}{c} 1+\frac{\left(b-1\right)}{q}s^{\prime \prime }n_{0}^{b-1}\int_{T_{i}}^{T_{M}}\exp\left(-\frac{E}{kT^{\prime }}\right)dT^{\prime }\\ =\left(\frac{\mathrm{bk}T_{M}^{2}}{\mathrm{qE}}\right)s^{\prime \prime }n_{0}^{b-1}\exp\left(-\frac{E}{kT_{M}}\right). \end{array}$$

It is important to note that Equations (1) and (3) are characterized by the following integral

(4) $$J=\int dT^{\prime }e^{-\frac{E}{kT^{\prime }}}.$$

Applying the integration by part method yields the following result

(5) $$J=Te^{-\frac{E}{\mathrm{kT}}}\sum_{n=1}^{\infty }n!{\left(-1\right)}^{n+1}{\left(\frac{\mathrm{kT}}{E}\right)}^{n}.$$

Due to the TL phenomenon typically occurring at temperatures below 800 K and energy values between 0.8 and 2 eV, this equation can be approximated by

(6) $$J=\left(\frac{kT^{2}}{E}\right)e^{-\frac{E}{\mathrm{kT}}}\left[1-\frac{2\mathrm{kT}}{E}\right].$$

Using this result along with Equation (3) in Equation (1) yields the following equation

(7) $$\begin{array}{c} I=I_{M}b^{\frac{b}{b-1}}\exp\left(\frac{E}{\mathrm{kT}}\frac{T-T_{M}}{T_{M}}\right){\left[z_{M}+\left(b-1\right)\left(1-\Delta \right)\frac{T^{2}}{T_{M}^{2}}\exp\left(\frac{E}{\mathrm{kT}}\frac{T-T_{M}}{T_{M}}\right)\right]}^{\frac{b}{1-b}}, \end{array}$$

where

(8) $$z_{M}=1+\Delta_{M}\left(b-1\right),$$

(9) $$\Delta =\frac{2\mathrm{kT}}{E},$$

(10) $$\Delta_{M}=\frac{2kT_{M}}{E}.$$

To determine the pre‐exponential factor, the following equation is used [15]:

(11) $$s^{\prime \prime }=\frac{1}{z_{M}}\frac{\mathrm{qE}}{kT_{M}^{2}}\exp\left(\frac{E}{kT_{M}}\right).$$

The “tgcd” package includes second derivative calculations to assist users in determining the number and position of peaks in the glow curve. The quality of fit is measured using the figure of merit (FOM) value as follows [14]

(12) $$\mathrm{FOM}=\sum_{i=1}^{i=n}\frac{\left|y_{i}-\hat{y_{i}}\right|}{A}\times 100,$$

where *y*_*i*_ is the *i*th observed value, $\hat{y_{i}}$ is the *i*th fitted value, *A* is the total area of the fit glow curve, and *n* is the number of fit data points [14].

### 2.6 Half‐Life Calculations

There are two ways to define the half‐life of TL traps [16, 17]. The first definition is the time required for the TL intensity *I*(*t*) of an isolated TL peak, to decrease to half of its initial value ($\tau_{1/2}$). The second definition is the time required for the total charge carriers trapped in a single trap *n*(*t*) to decay to half of its initial value ($t_{1/2}$). For obtaining an expression for the second definition, the GOK equation becomes as follows

(13) $$\frac{\mathrm{dn}}{n^{b}}=-s^{\prime \prime }\exp\left(-\frac{E}{kT_{s}}\right)\mathrm{dt},$$

where *T*s is the storage temperature.

After integration, this equation becomes

(14) $$t_{1/2}=\left(\frac{1}{2^{1-b}}-1\right)\frac{n_{0}^{1-b}\exp\left(\frac{E}{kT_{s}}\right)}{s^{\prime \prime }\left(b-1\right)}.$$

By applying Equation (11), Equation (14) transforms into [16, 17, 18]

(15) $$\begin{array}{c} t_{1/2}=\left(1-\frac{1}{2^{1-b}}\right)\left(\frac{kT_{m}^{2}}{\mathrm{qE}}\right)\frac{\exp\left[\frac{E}{k}\left(\frac{1}{T_{s}}-\frac{1}{T_{m}}\right)\right]}{\left(1-b\right)}\left(1+\frac{2kT_{m}\left(b-1\right)}{E}\right), \end{array}$$

This equation enables the determination of the half‐life when *b*, *T* ₘ, *T* ₛ, and *E* are known parameters.

## 3 Results and Discussion

### 3.1 Structural and Elemental Characterization

The XRD pattern confirmed the wurtzite phase of AlN with lattice parameters *a* = 3.111 and *c* = 3.978 Å (JCPDS card No. 03‐1169). The EDS spectrum showed characteristic X‐ray peaks corresponding to Al, N, C, O, Si, and Fe, indicating the presence of these elements in the synthesized product [8]. Table 1 summarizes the results of the EDS analysis.

**TABLE 1:** SEM‐EDS results for AlN.

Element	wt.%	at. %
Nitrogen (N)	40.56	52.64
Aluminum (Al)	46.64	31.43
Carbon (C)	5.37	8.13
Oxygen (O)	6.53	7.42
Silicium (Si)	0.22	0.14
Iron (Fe)	0.36	0.12
Others	0.32	0.12

**FIGURE 1:**
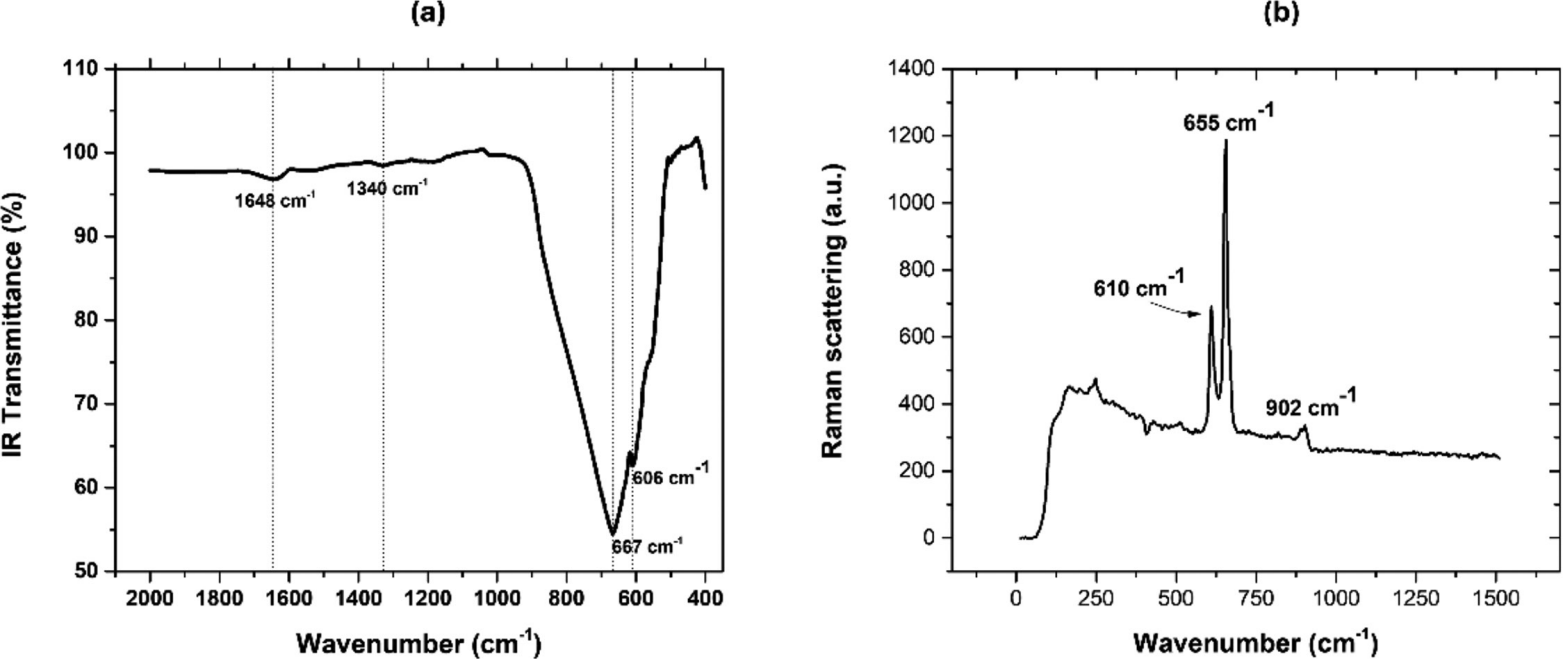
(a) FTIR and (b) Raman spectra of AlN powders.

Figure 1a presents the FTIR spectrum, showing characteristics of the transmittance minima of AlN at 606, 667, and 1340 cm^−1^. The first and second peaks correspond to the transversal optical modes *A*_1_(TO) and E1 (TO), respectively [19]. The third peak corresponds to the longitudinal optical mode Al‐N (LO) [3]. The peak observed at 1648 cm^−1^ corresponds to the vibrational mode characteristic of H‐O‐H and could be associated with the hygroscopic properties of the material [3]. Figure 1(b) displays the Raman scattering spectrum, showing three scattering maxima at 610, 655, and 902 cm^−1^ corresponding to A1(TO), $E_{2}^{2}$, and *A*_1_(LO) phonon symmetries, respectively [19]. FTIR and Raman results are summarized in Table 2.

**TABLE 2:** FTIR and Raman results for AlN.

Phonon symmetry	Phonon energy (cm^−1^)	FTIR phonon energy (cm^−1^)	Raman phonon energy (cm^−1^)
$E_{2}^{2}$	657	—	655
$A_{1}\left(\text{TO}\right)$	611	606	610
$E_{1}\left(\text{TO}\right)$	666	667	—
$A_{1}\left(\mathrm{LO}\right)$	890	—	902

These results show that the synthesis of AlN by the NH₄Cl(s)‐assisted vapor phase reaction method is suitable for the produce AlN in the hexagonal wurtzite phase without formation of additional phases. Furthermore, this method facilitates the incorporation of various unintentional impurities, which can increase the formation of crystal defects. These defects can act like traps and recombination centers, thus promoting TL emission.

### 3.2 Photoluminescence

**FIGURE 2:**
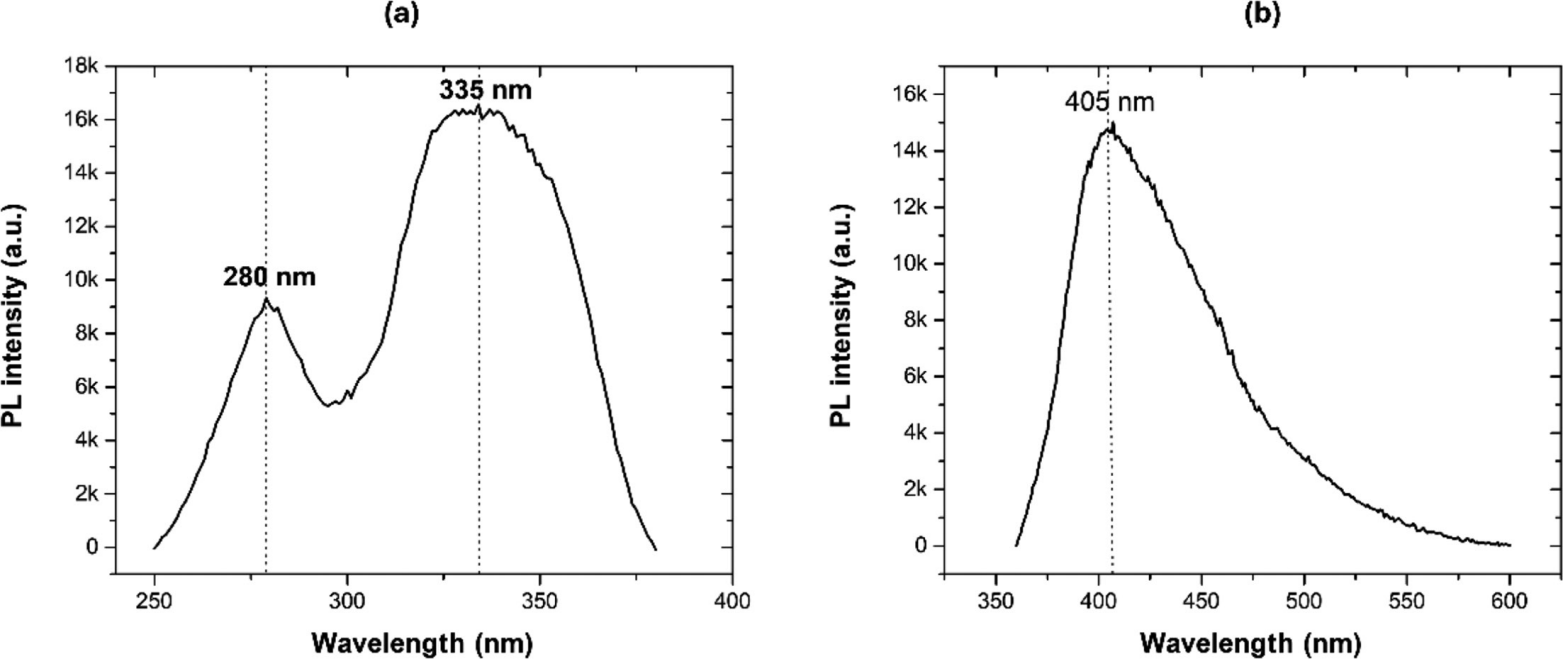
The photoluminescence (a) excitation and (b) emission spectra of AlN powders.

Figure 2a shows the PL excitation spectrum recorded at 405 nm emission for AlN, which exhibits two excitation peaks at 280 and 335 nm. The PL excitation region centered at 280 nm is consistent with the PL band from C_N_Si_Al_ complex defects calculated by Aleksandrov and Zhuravlev [12]. The second PL region centered at 335 nm could be associated with the PL band from both V_Al_3O_N_ and C_N_V_N_ complex defects predicted at 340 nm by Aleksandrov and Zhuravlev [12]. Figure 2b shows the PL emission spectrum under 335 nm excitation. This spectrum exhibits a complex shape with a predominant emission peak at 405 nm, which agrees with the PL emissions reported by other authors [9, 10]. This PL peak could be associated with V_Al_2O_N_ [12].

### 3.3 Thermoluminescence

**FIGURE 3:**
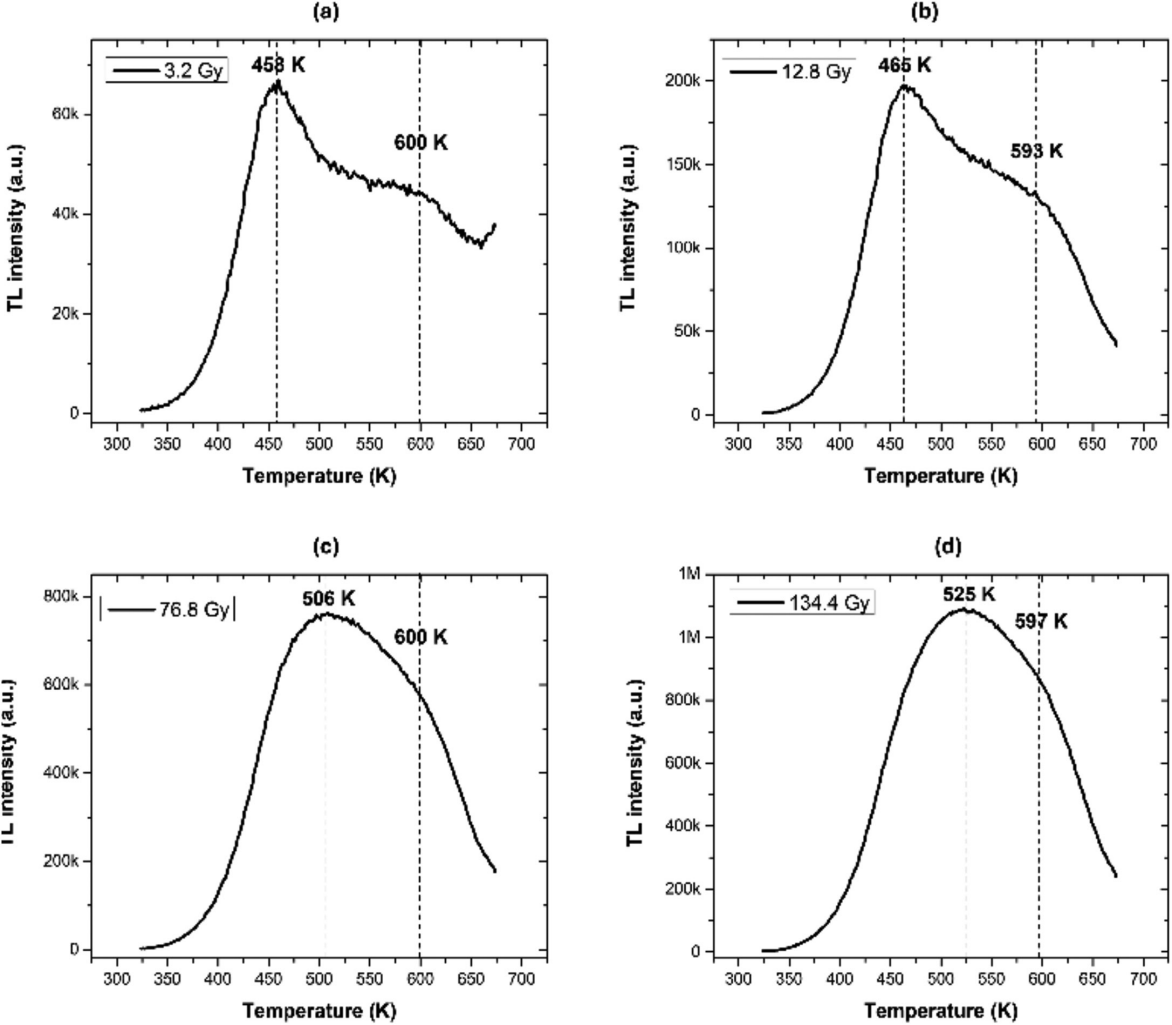
AlN glow curves obtained at different alfa/gamma‐doses. (a) and (b) low doses (3.2 and 12.8 Gy, respectively), and (c) and (d) high doses (76.8 and 134.4 Gy, respectively).

**FIGURE 4:**
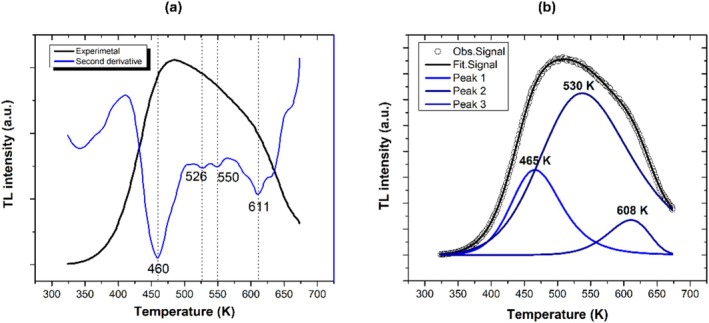
(a) Second derivative and (b) deconvolution of TL glow curve.

Figure 3 shows the AlN glow curves obtained at different alpha/gamma doses. These glow curves show two peaks at 460 and 600 K at low doses, while at high doses, they show an additional peak at 525 K. This suggests that the glow curve could be constituted by three peaks. Figure 4a shows the second derivative of the glow curve, which suggests four peaks at 460, 526, 550, and 611 K. Finally, Figure 4b shows the results of the deconvolution method, which fits three peaks to the glow curve at 465, 530, and 608 K.

Table 3 presents the results obtained for activation energy (*E*), the kinetics order (*b*), frequency factor (*s*), and area for each peak by the deconvolution method. These results were used to determine the half‐life for each peak. From this table, it can be inferred that 71% of the TL signal fades after 2.23 days. The activation energies obtained for the first and second peaks (0.69 and 0.45 eV, respectively) agree with the values reported by Shulz et al. for Si_Al_ defect (0.5 eV) [20]. Low temperatures and low activation energies for TL centers are related to short half‐lives, which agrees with values reported in Table 3 for TL Peaks 1 and 2 (2.23 and 0.82 days, respectively).

**TABLE 3:** Kinetic parameters obtained by package “tgcd” in R deconvolution method.

Parameter	Peak 1	Peak 2	Peak 3
Kinetic order	1.95 ± 0.10	2.00 ± 0.01	1.14 ± 0.13
Frequency factor (s^−1^)	⁓10^5^–10^6^	⁓10^2^–10^3^	⁓10^7^–10^8^
Activation energy (eV)	0.69 ± 0.03	0.45 ± 0.02	1.06 ± 0.07
Temperature (K)	461 ± 5	531 ± 4	610 ± 3
Half‐life	2.23 d	0.82 d	522.4 y
Area (%)	24.1	69.6	6.3

**FIGURE 5:**
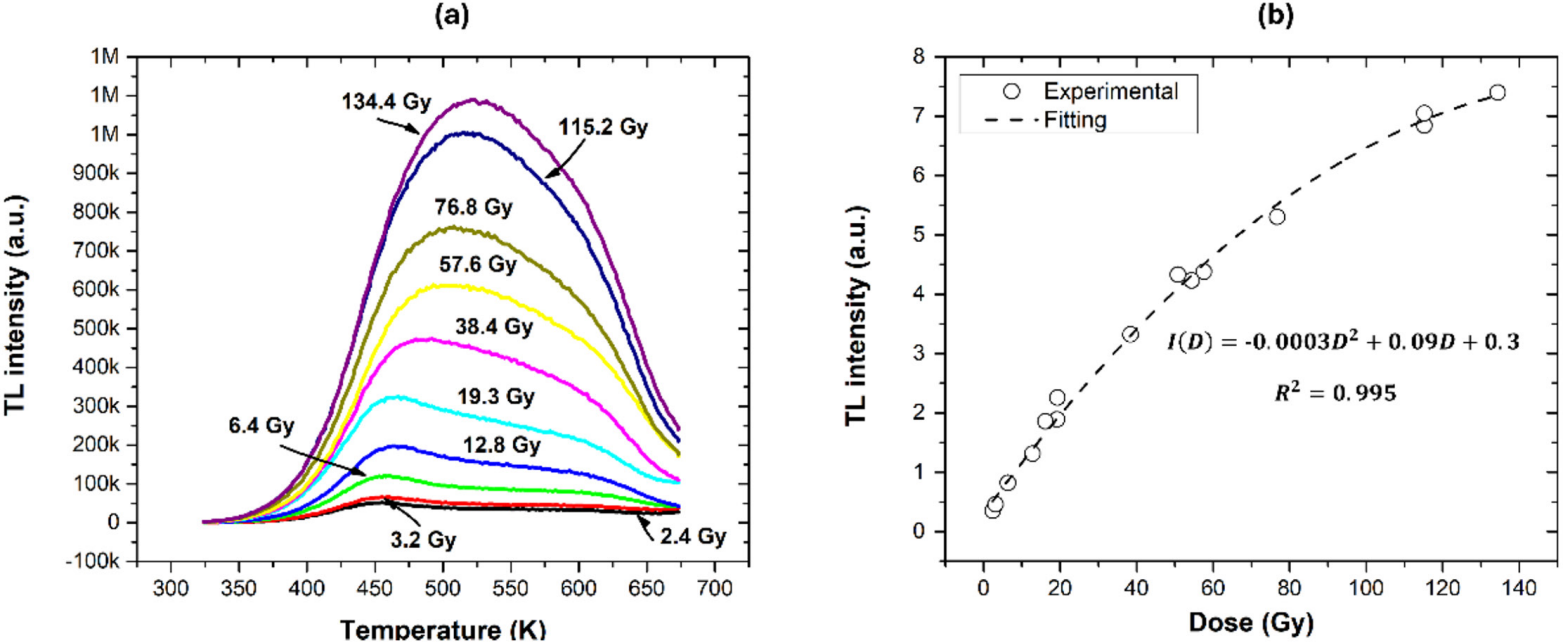
(a) Evolution curves and (b) TL response as a function of dose for whole TL curves.

**FIGURE 6:**
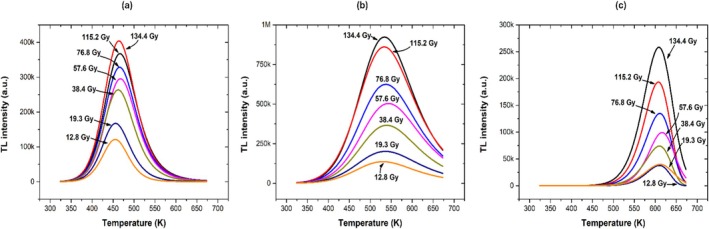
Glow curve evolution as a function dose for each TL component in the glow curve. (a) First peak, (b) second peak, and (c) third peak.

**FIGURE 7:**
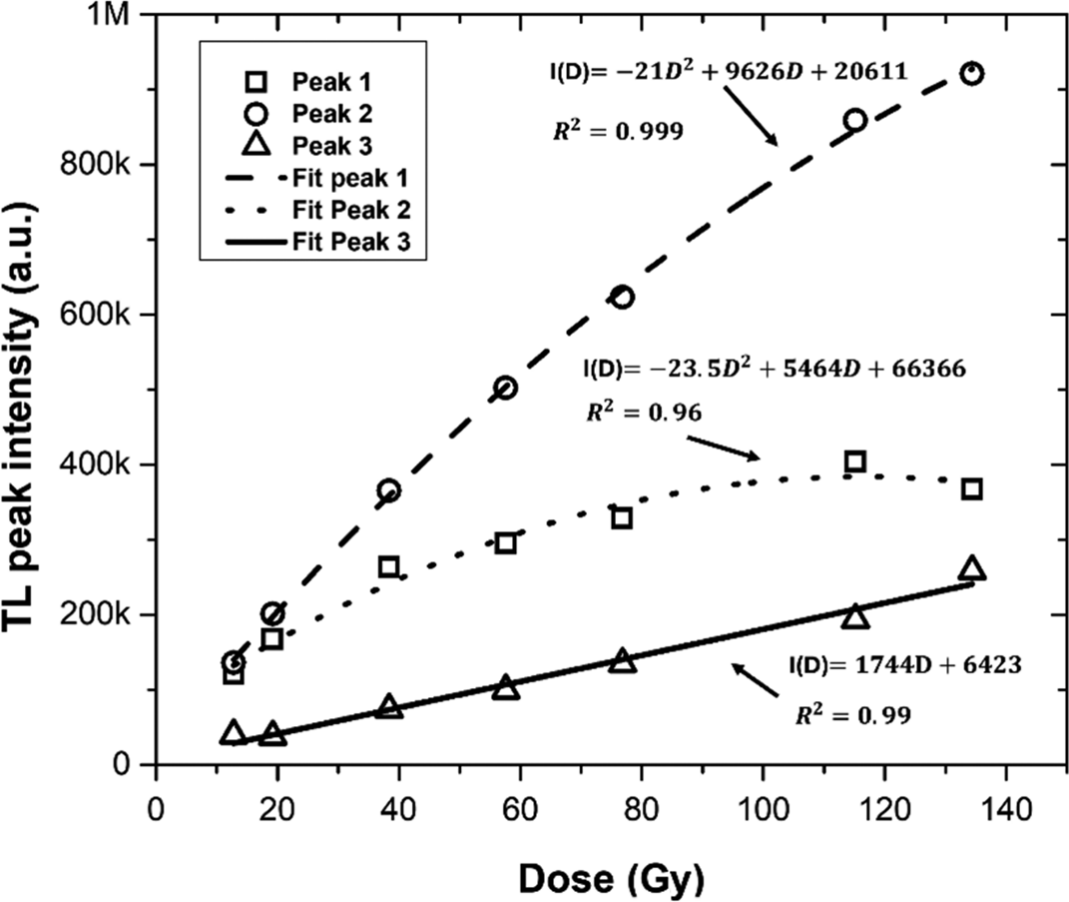
TL maximum intensity as a function of dose for each glow peak.

Figure 5a shows the evolution of the glow curve as a function of dose. Figure 5b shows the TL response (total area) as a function of dose, which presents a saturation behavior at about 140 Gy. Figures 6a,b,c shows the evolution of each glow peaks. Figure 7 shows the TL maximum intensity as a function of dose for each glow peak. Both the first and second peaks show a tendency to saturation, while the third peak exhibits a linear behavior in the range from 12.6 to 136 Gy.

## 4 Conclusions

In conclusion, the results show that the glow curve of AlN doped with oxygen (O), carbon (C), silicon (Si), and iron (Fe) irradiated with alpha/gamma radiation are composed by three peaks centered at 465, 530, and 608 K, with half‐lives of 2.23 days, 0.82 day, and 522.4 years, respectively. These results indicate that only 29% of the TL signals will remain in the centers after 2.23 days. PL and TL results suggest a connection between the TL peaks centered at 465 and 530 K and C_N_Si_Al_ complex defects. Deconvolution results show that the peak centered at 608 K has suitable properties for potential applications in TL dosimetry. Further investigations must be conducted on AlN doped with Si, O, or C at different concentrations or combinations to reduce the high fading observed in this material.

## Data Availability

Research data are not shared.

## References

[1] K. Kojima , G. Okada , K. Fukuda , and T. Yanagida , “Influence of SrF_2_‐Doping in AlN Ceramics on Scintillation and Dosimeter Properties,” Radiation Measurements 94 (2016): 78–82.

[2] E. P. J. Merkx , T. G. Lensvelt , and E. van der Kolk , “Solar Energy Mater,” Solar Cells 200 (2019): 110032.

[3] M. Zheng , Q. Jia , S. Zhu , and X. Liu , “Large Scale Synthesis and Photoluminescent Property of Ultra‐Long AlN Nanowires via a NH_4_Cl Assisted Chemical Vapor Reaction Method,” Ceramics International 44 (2018): 7267–7272.

[4] M. Benabdesselam , P. Iacconi , D. Lapraz , P. Grosseau , and B. Guilhot , “Thermoluminescence of AlN. Influence of Synthesis Processes,”,Journal of Physical Chemistry 99 (1995): 10319–10323.

[5] T. Honma , Y. Kuroki , T. Okamoto , et al., “Transmittance and Cathodoluminescence of AlN Ceramics Sintered With Ca3Al2O6 as Sintering Additive,” Ceramics International 34 (2008): 943–946.

[6] I. A. Aleksandrov , V. G. Mansurov , V. F. Plyusnin , and K. S. Zhuravlev , “Time‐Resolved Photoluminescence Characterization of 2 eV Band in AlN,” Physica Status Solidi C: Current Topics in Solid State Physics 12 (2015): 353–356.

[7] Y. Onoda , H. Kimura , T. Kato , K. Fukuda , N. Kawaguchi , and T. Yanagida , “Thermally Stimulated Luminescence Properties of Eu‐Doped AlN Ceramic,” Optik 181 (2019): 50–56.

[8] R. Martínez‐Baltezar , J. Azorín‐Nieto , R. Martinez‐Baltazar , and E. Cortés‐Ortiz , “Thermoluminescent Characteristics of UV‐Irradiated Aluminum Nitride (AlN),” Applied Radiation and Isotopes 200 (2023): 110977. 37595322 10.1016/j.apradiso.2023.110977

[9] L. Trinkler , P. Christensen , N. A. Larsen , and B. Berzina , “Thermoluminescence Properties of AlN Ceramics,” Radiation Measurements 29 (1998): 341–348.

[10] T. Yanagida , Y. Fujimoto , N. Kawaguchi , and S. Yanagida , “Dosimeter Properties of AlN,” Journal of the Ceramic Society of Japan 121 (2013): 988–991.

[11] L. Trinkler , B. Berzina , A. Auzina , M. Benabdesselam , and P. Iacconi , “UV Light Energy Storage and Thermoluminescence in AlN Ceramics,” Physica Status Solidi C: Current Topics in Solid State Physics 4 (2007): 1032–1035.

[12] I. A. Aleksandrov and K. S. Zhuravlev , “Luminescence Line Shapes of Band to Deep Centre and Donor–Acceptor Transitions in AlN,” Journal of Physics. Condensed Matter 32 (2020): 435501. 10.1088/1361-648X/aba295 32620002

[13] J. Peng , Z. Dong , and F. Han , “tgcd: An R Package for Analyzing Thermoluminescence Glow Curves,” Software X 5 (2016): 112–120.

[14] J. Peng , G. Kitis , A. M. Sadek , E. C. K. Asal , and Z. Li , “Thermoluminescence Glow‐Curve Deconvolution Using Analytical Expressions: A Unified Presentation,” Applied Radiation and Isotopes 168 (2021): 109440. 33268224 10.1016/j.apradiso.2020.109440

[15] G. Kitis , J. M. Gomez‐Ros , and J. W. Tuyn , “Thermoluminescence Glow‐Curve Deconvolution Functions for First, Second and General Orders of Kinetics,” Journal of Physics D: Applied Physics 31 (1998): 2636–2641.

[16] V. Pagonis , G. Kitis , and G. S. Polymeris , “On the Half‐Life of Luminescence Signals in Dosimetric Applications: A Unified Presentation,” Physica B 539 (2018): 35–43.

[17] J. Azorín‐Nieto , C. Furetta , E. Ortiz‐Martínez , and C. Azorin‐Vega , “Calculation of the Half Life for the Thermoluminescent Signal of Beryllium Oxide,” Applied Radiation and Isotopes 186 (2022): 110291. 35617892 10.1016/j.apradiso.2022.110291

[18] C. Furetta and G. Kitis , “Models in thermoluminescence,” Journal of Materials Science 39 (2004): 2277–2294.

[19] T. Prokofyeva , M. Seon , J. Vanbuskirk , et al., “Vibrational Properties of AlN Grown on (111)‐Oriented Silicon,” Physical Review B 63 (2001): 125313.

[20] T. Schulz , K. Irmscher , M. Albrecht , C. Hartmann , J. Wollweber , and R. Fornari , “n‐Type Conductivity in Sublimation‐Grown AlN Bulk Crystals,” Physica Status Solidi RRL: Rapid Research Letters 1 (2007): 147–149.

